# Impact of Depression on Postoperative Medical and Surgical Outcomes in Spine Surgeries: A Systematic Review and Meta-Analysis

**DOI:** 10.3390/jcm13113247

**Published:** 2024-05-31

**Authors:** Sepehr Aghajanian, Arman Shafiee, Mohammad Mobin Teymouri Athar, Fateme Mohammadifard, Saba Goodarzi, Fatemeh Esmailpur, Aladine A. Elsamadicy

**Affiliations:** 1 Student Research Committee, School of Medicine, Alborz University of Medical Sciences, Karaj 3198764653, Iran; sepehraghajanian2@gmail.com (S.A.); f.esmaeilpur@gmail.com (F.E.); 2 Neuroscience Research Center, Iran University of Medical Sciences, Tehran 14496-14535, Iran; 3 School of Medicine, Shahid Beheshti University of Medical Sciences, Tehran 19839-63113, Iran; 4 Department of Neurosurgery, Yale University School of Medicine, New Haven, CT 06520, USA

**Keywords:** depression, spinal surgery, complication, prognosis, psychiatric disorder, spinal fusion, thoracolumbar surgery

## Abstract

**Introduction:** The relationship between psychiatric disorders, including depression, and invasive interventions has been a topic of debate in recent literature. While these conditions can impact the quality of life and subjective perceptions of surgical outcomes, the literature lacks consensus regarding the association between depression and objective perioperative medical and surgical complications, especially in the neurosurgical domain. **Methods:** MEDLINE (PubMed), EMBASE, PsycINFO, and the Cochrane Library were queried in a comprehensive manner from inception until 10 November 2023, with no language restrictions, for citations investigating the association between depression and length of hospitalization, medical and surgical complications, and objective postoperative outcomes including readmission, reoperation, and non-routine discharge in patients undergoing spine surgery. **Results:** A total of 26 articles were considered in this systematic review. Upon pooled analysis of the primary outcome, statistically significantly higher rates were observed for several complications, including delirium (OR:1.92), deep vein thrombosis (OR:3.72), fever (OR:6.34), hematoma formation (OR:4.7), hypotension (OR:4.32), pulmonary embolism (OR:3.79), neurological injury (OR:6.02), surgical site infection (OR:1.36), urinary retention (OR:4.63), and urinary tract infection (OR:1.72). While readmission (OR:1.35) and reoperation (OR:2.22) rates, as well as non-routine discharge (OR:1.72) rates, were significantly higher in depressed patients, hospitalization length was comparable to non-depressed controls. **Conclusions:** The results of this review emphasize the significant increase in complications and suboptimal outcomes noted in patients with depression undergoing spinal surgery. Although a direct causal relationship may not be established, addressing psychiatric aspects in patient care is crucial for providing comprehensive medical attention.

## 1. Introduction

Depression is a complex and multifaceted mental disorder, which is distinguished by enduring feelings of sadness, despair, and a diminished interest in activities that were once pleasurable [1]. Its impact extends beyond the emotional realm, influencing physical health and potentially intersecting with the outcomes of various medical interventions. Depression can lead to a wide array of physical problems and increase the risk for or worsen certain physical illnesses or conditions [2]. A significant proportion of patients complaining of symptoms such as lower back pain need to undergo different types of spine surgeries. A diverse array of procedures such as laminectomy, discectomy, kyphoplasty, and lumbar spinal fusion address a spectrum of spinal disorders. Within the surgical context, depression has been linked to unfavorable postoperative results, such as heightened morbidity and mortality rates [3]. Interestingly, previous research has shown that patients with spinal deformities, specifically, have demonstrated a heightened susceptibility to depression when compared to the broader population [4]. Moreover, there is evidence suggesting that depression adversely impacts patient outcomes following lower back surgery [5,6]. Nevertheless, there is a scarcity of research investigating the effects of comorbid psychiatric conditions on different postoperative outcomes such as perioperative adverse events, surgical site and systemic infections, length of stay in the hospital, and surgical and medical readmission. Understanding the influence of depression on the clinical outcomes of spine surgeries is crucial since a deeper understanding of this relationship can inform the development of targeted perioperative strategies, with the potential to reduce complications and enhance overall patient care and satisfaction. Although the expected correlation of preoperative depression with postoperative self-reported quality of life and patient disability outcomes in spinal surgeries have been recently investigated [7], the impact of depression on medical and surgical outcomes is not thoroughly understood and is further explored in a quantitative manner in this review.

## 2. Materials and Methods

### 2.1. Protocol and Registration

This systematic review and meta-analysis adhered to the Preferred Reporting Items for Systematic Reviews and Meta-Analyses (PRISMA) guidelines [8]. The review protocol was registered with PROSPERO (CRD42024513255).

### 2.2. Eligibility Criteria

Studies were included if they met the following criteria: (1) peer-reviewed articles published in English; (2) studies that included patients undergoing spinal surgery; (3) studies that assessed preoperative depression status in patients undergoing spine surgery; and (4) studies that reported on postoperative surgical and medical outcomes, including but not limited to mortality, non-routine discharge, overall complications, readmission, reoperation, and length of stay. Excluded were case reports, conference abstracts, reviews, and studies not reporting on the specific outcomes of interest. Studies restricted to reporting self-reported quality of life and disability questionnaires were also excluded.

### 2.3. Search Strategy and Data Collection

A comprehensive search was conducted in MEDLINE (PubMed), EMBASE, PsycINFO, and the Cochrane Library from inception until 10 November 2023, with no language restrictions. The following search strategy was used: (Depress* OR Depressive neurosis) AND ((Spin* surgery) OR SCS OR (Spinal cord surgery) OR (Lumbar Surgery) OR Kyphoplasty OR Vertebroplasty OR (Vertebral column resection) or VCR) AND (Outcome OR prognos* OR function OR readmission OR reoperation OR complication OR Mortality OR death OR quality). Reference lists of included studies and relevant reviews were screened to identify additional studies.

Two reviewers independently screened titles and abstracts to determine eligibility. Full texts of potentially eligible studies were then retrieved and assessed for inclusion by the same reviewers. Any discrepancies were resolved through discussion or consultation with a third reviewer. A standardized data extraction form was employed to gather information from each included study regarding study characteristics (author, year, country), participant characteristics (sample size, age), details of the spinal surgery, methods of depression assessment, and postoperative outcomes. Data extraction was performed independently by two reviewers, with discrepancies resolved by consensus or involvement of a third reviewer.

The quality of included studies was assessed using the Newcastle–Ottawa assessment tool [9]. Similarly, two reviewers independently assessed the risk of bias, with any discrepancies resolved through discussion or consultation with a third reviewer.

### 2.4. Data Synthesis and Analysis

A quantitative synthesis was conducted using aggregated participant data, requiring at least two studies for outcome analysis. A random-effects model was employed to account for the varying designs and populations of the included studies. Secondary subgroup analysis was performed to distinguish deep from superficial surgical site infections and to categorize readmission rates based on the assessment interval. Study heterogeneity was assessed with the I^2^ or Q test. Effect measures were calculated for odds ratios and mean ratios. The primary outcome was the number of participants experiencing adverse events or medical/clinical complications, as defined by each study. Secondary outcomes included the length of hospital stay, non-routine discharge, readmission, reoperation, and mortality rate. Each outcome was analyzed through a generic inverse variance meta-analysis to pool studies on individual complications. The measure of effect for binary outcomes was chosen as odds ratios, either calculated via contingency tables or directly extracted from the studies. The standard error of the odds ratios reported in each study were back-calculated using the 95% confidence intervals in Stata Statistical Software (Release 17, College Station, TX, USA: StataCorp LLC).

## 3. Results

**Figure 1:**
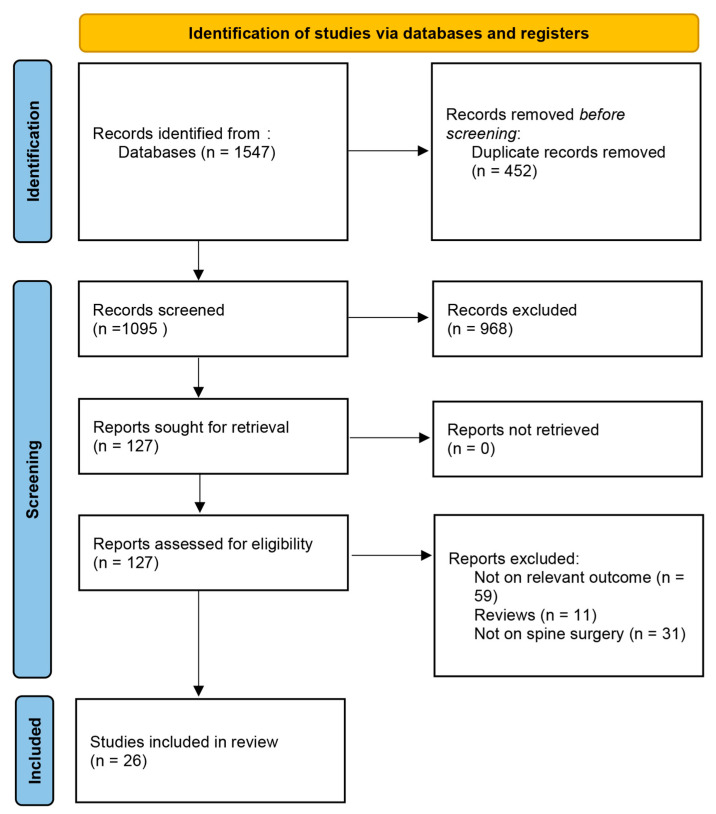
PRISMA flowchart illustrating the study screening and inclusion process.

The initial search yielded 1547 citations, out of which 127 underwent full-text screening (Figure 1). In total, 26 were included in this systematic review [10,11,12,13,14,15,16,17,18,19,20,21,22,23,24,25,26,27,28,29,30,31,32,33,34,35]. While most of the studies were rated high in terms of quality as assessed by the Newcastle–Ottawa scale (Supplementary Materials), the majority failed to adequately define and describe the diagnostic criteria for depression in the included studies. The majority of studies included patients undergoing surgery for degenerative spinal diseases (Table 1). 

Studies reported various postoperative outcomes, including the rates of adverse events (such as surgical site and nonsurgical infections, and cardiovascular complications), non-routine discharges (to rehabilitation facilities or with external services), hospitalization duration, readmissions, and reoperation rates. The number of studies evaluating mortality was insufficient for a quantitative analysis. Although not selected as pooled outcomes, Huang et al. and Schoell et al. found higher rates of ventilator use and failed back surgery syndrome, respectively [25,31]. Moreover, Holbert et al. identified a higher risk of short-term emergency visits, readmissions, and complications. However, their study was excluded from this meta-analysis due to the concurrent inclusion of patients with anxiety [24].

### 3.1. Primary Outcome

The meta-analysis revealed higher odds of multiple complications in patients with depression (Table 2). The pooled data demonstrated a significant association between depression and postoperative delirium (Odds Ratio [OR]: 1.91, 95% CI: [1.77–2.07]), more than threefold increased odds of deep vein thrombosis (OR: 3.72, 95% CI: [1.03–13.42]), a sixfold increase in fever risk (OR: 6.34, 95% CI: [1.03–13.42]), a fourfold increase in hematoma formation (OR: 4.70, 95% CI: [1.44–15.38]) and hypotension (OR: 4.32, 95% CI: [1.88–9.95]), and a higher risk of pulmonary embolism (OR: 3.79, 95% CI: [1.21–11.92]), sensory deficits (OR: 6.02, 95% CI: [2.55–14.20]), weakness (OR: 6.52, 95% CI: [3.54–12.01]), and surgical site infections (OR: 1.36, 95% CI: [1.32–1.40]). Subgroup analyses for deep versus superficial surgical site infections did not reveal a significant pattern between the subtypes of infection and depression. Urinary tract complications were also more prevalent in patients with depression, including urinary tract infections (OR: 1.72, 95% CI: [1.03–2.88]) and urinary retention (OR: 4.63, 95% CI: [2.11–10.14]).

Although there was a trend toward statistical significance, the rates of sepsis/infection, myocardial infarction, and pneumonia were not distinctly different between the two groups (see Supplementary Materials).

### 3.2. Secondary Outcomes

**Figure 2:**
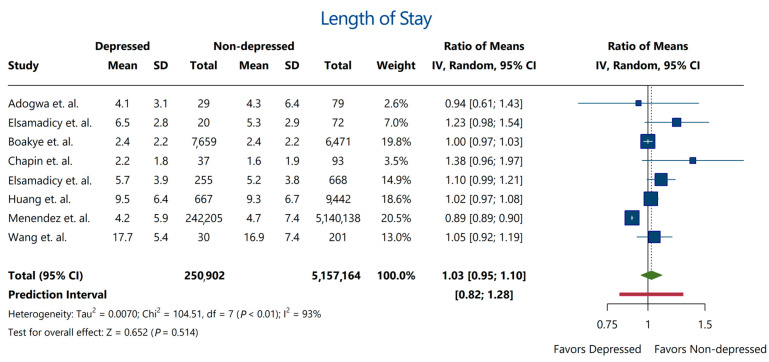
Length of hospitalization between depressed and non-depressed patients. The analysis revealed a negligible (3%) increase in hospitalization duration that was statistically insignificant. Red bar indicates the prediction interval of the estimate. Estimates were derived from studies by Adogwa et al. [10], Elsamadicy et al. [19], Boakye et al. [13], Chapin et al. [14], Elsamadicy et al. [18], Huang et al. [25], Menendez et al. [29], and Wang et al. [34].

Despite the association between depression and various complications and secondary outcomes, the majority of included studies showed a consensus that hospitalization length did not differ significantly between depressed and non-depressed patients (Ratio of means: 1.03; 95% CI: [0.95–1.10]) (Figure 2).

**Figure 3:**
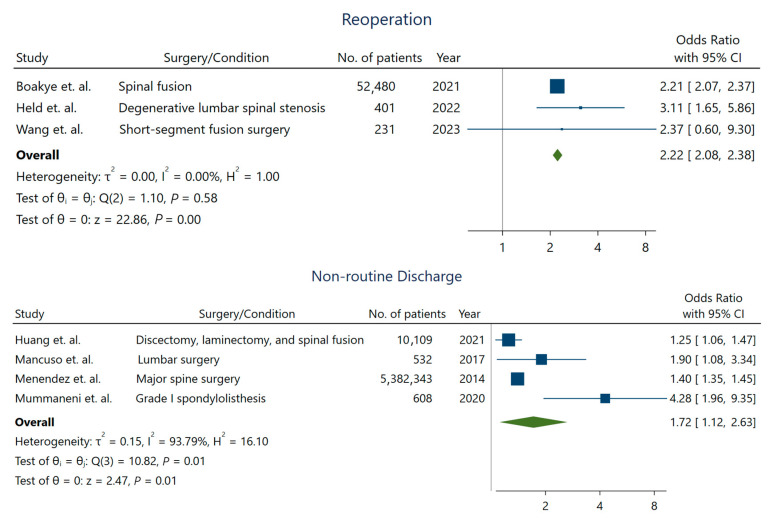
Reoperation and non-routine discharge rate in patients with depression. For reoperation outcome, estimates were derived from Boakye et al. [13], Held et al. [23], and Wang et al. [34]. The estimate of the non-routine discharge outcome was pooled from the studies by Huang et al. [25], Mancuso et al. [28], Menendez et al. [29], and Mummaneni et al. [30].

**Figure 4:**
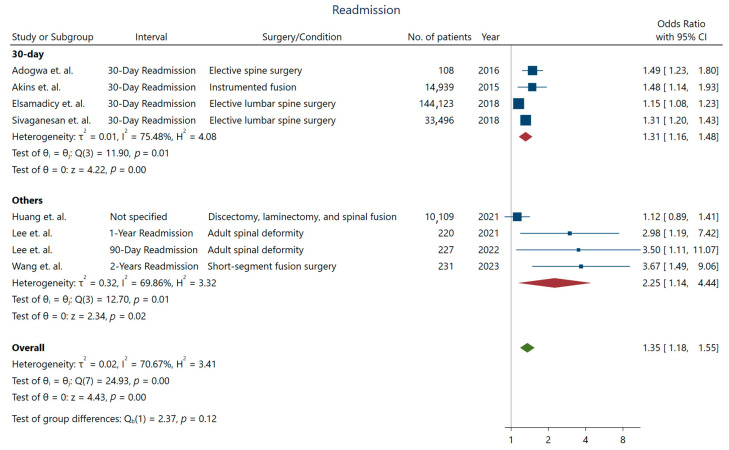
Short and long-term readmission rate in patients with depression. Data derived from Adogwa et al. [10], Akins et al. [11], Elsamadicy et al. [20], Sivaganesan et al. [32], Huang et al. [25], Lee et al. [26,27], and Wang et al. [34].

While the length of stay was not longer for depressed patients undergoing spine surgery, we observed a significant increase in the rates of non-routine discharge (OR: 1.72; 95% CI: [1.12–2.63]) (Figure 3), higher readmission rates (OR: 1.35; 95% CI: [1.18–1.55]), and reoperation (OR: 2.22; 95% CI: [2.08–2.38]) in these patients [10]. Studies with longer intervals for assessing readmission were more likely to detect a higher association between depression and readmission rates (Figure 4). 

## 4. Discussion

The World Health Organization considers major depressive disorder as the fourth leading cause of morbidity, which is projected to become the second leading cause of disability by 2030 [36]. The long-term implications of mental health on surgical outcomes highlight the imperative need for integrated mental health assessments and targeted interventions to mitigate the adverse effects of depression and enhance overall patient care. The findings of our study underscore the significant impact of depression on the clinical outcomes of spine surgeries. Patients with depression exhibited a heightened susceptibility to a range of complications, including delirium, deep vein thrombosis (DVT), fever, hematoma, hypotension, sensory deficit, surgical site infection, urinary retention, and weakness and a higher likelihood of hospital readmission, extended length of hospital stay and a twofold increased risk of reoperation among depressed patients suggesting a slower and potentially more challenging recovery process. 

Although the causal association between these factors was not explored in the studies included in this review, the literature offers intriguing insights into the relationship between depression and surgical outcomes through various interconnected mechanisms. Preoperative depression may compromise patients’ motivation for engaging in essential physical and social activities crucial for maintaining or regaining functional capacity. This diminished motivation often translates into challenges in effectively participating in physical therapy, a cornerstone for functional recovery after spine surgery. Deteriorating baseline health status in these patients can be further exacerbated by higher rate of comorbid disorders. Indeed, Poorman et al. reported higher rate of osteoporosis, rheumatoid arthritis, and connective tissue disorders in patients undergoing surgery for cervical deformity [37]. Furthermore, depressive symptoms can influence patients’ adherence to prescribed medical regimens, affecting their biological milieu through neuroendocrine and inflammatory mechanisms [38,39]. This not only impedes the healing process but may also contribute to heightened susceptibility to complications.

Depression may also significantly influence a patient’s perception of self-efficacy, limiting their active involvement in medical care, rehabilitation programs, and social activities—all integral components for optimizing functional recovery post-spine surgery. Beyond behavioral and psychological aspects, the intricate relationship between depression and the immune system, including the pro-inflammatory milieu caused by psychological stress, diminished T-cell response and the shift away from Thelper-1 phenotype, and the immunosuppressive effects of certain antidepressant medications [3,40,41], may elaborate on the increased risk of postoperative SSI and systemic infections. Additionally, epidemiological studies indicate that mood disorders are linked to an increased body mass index, hypertension, elevated cholesterol levels, diabetes, lack of physical activity, and consistent engagement in smoking and nicotine dependence [42]. Understanding these multifaceted pathways is essential for devising comprehensive strategies to address preoperative depression and enhance overall patient outcomes in the surgical setting.

Two preceding systematic reviews have investigated the correlation between preoperative depression and the outcomes of spine surgeries, offering diverse perspectives on this intricate interconnection. One review posits that the degree of improvement is similar when comparing groups with and without depression; however, individuals with depression reported a more pronounced level of pain [7]. Notably, our research deviates by scrutinizing distinct, more objectively measurable results. The review conducted by Javeed et al. asserts a direct link between preoperative depression and unfavorable quality of life and disability outcomes [7]. Mollon et al. [43] and Häuser et al. [44] independently validated that preoperative depression significantly associates with increased rates of postoperative complications. Additionally, Mollon et al. underscored that individuals with a history of depression are more prone to postoperative adverse events such delirium, infections, and anemia necessitating blood transfusion. Interestingly, there was no statistically significant difference in in-hospital death rates. On the other hand, depressed patients had slightly prolonged hospital stays and had a higher likelihood of non-routine discharge. Furthermore, Adogwa et al. [10] identified a correlation between depression and the 30-day readmission rate. Another cohort study by Chaichana et al. [45] affirmed the impact of preoperative depression and heightened somatic awareness on the probability of achieving clinically meaningful improvements in disability or quality of life. These cumulative findings underscore the intricate and multifaceted nature of the association between preoperative depression and outcomes in spine surgeries.

The profound impact of depression on surgical outcomes enables the creation of collaborative care models, uniting surgical teams, anesthesiologists, and mental health professionals to optimize patient preparation and facilitate a more holistic recovery process [18,46]. While short-term interventions in the perioperative may not significantly reduce depressive symptomatology, targeted psychosocial interventions for high-risk patients identified using concise screening tools like the 9-item Patient Health Questionnaire-9 (PHQ-9) and the 14-item Hospital Anxiety and Depression Scale (HADS) will not only enable better assessment of postoperative complication risks but also positively impact surgical outcomes. Recent literature indicates that this approach may have successfully reduced adverse outcomes, including medical complications, even though the rate of readmission remained similar. This is based on a retrospective study examining the impact of preoperative depression screening and psychotherapy visits for patients with a history of depression undergoing short-segment lumbar fusion [47]. Moreover, the heightened risk in patients with depressive phenotypes may be secondary to poor overall health linked to impaired mental well-being, rather than the psychosomatic manifestation of the disease. This necessitates a more rigorous approach to preoperative testing for spinal surgery in these subsets of patients. 

Acknowledging several limitations in this review is crucial. Despite a significantly higher risk of complications, the lack of association between depression and adverse events may be attributed to varying definitions and the limited number of studies focusing on distinct complications. Additionally, the discrepancy could stem from clustered complications in depressed patients, leading to a similar overall complication rate but a higher comorbidity rate. Data scarcity comparing medicated and non-medicated depressed patients hinders comprehensive conclusions. Moreover, the absence of detailed information on surgery-related factors and comorbid conditions introduces potential confounding variables. Heterogeneous depression screening methods and the inclusion of various surgeries add result variability. Lastly, the predominance of cohort studies underscores the need for cautious interpretation. Nevertheless, the need for further research is evident. Future studies with a focus on specific types of surgeries and a larger cohort of patients are imperative to refine our insights and enhance the precision of interventions. This study serves as a stepping stone, emphasizing the ongoing importance of exploring the nuanced interplay between mental health and surgical outcomes for the advancement of patient care strategies in diverse surgical contexts.

## Figures and Tables

**Table 1:** Description and summary of the included studies.

No.	Author	Country	No. of Participants	Mean Age (SD)	Operation	Depression Criteria	Evaluated Outcomes	Follow-Up Period
1	Adogwa et al. [10]	USA	108	Depressed cohort: 58.2 ± 10.4 Non-depressed cohort: 57.0 ± 14.6	Elective spine surgery	Physician-diagnosed	Readmission, surgical site infection (SSI), length of stay (LOS), pneumonia, infection, myocardial infarction (MI), pulmonary embolism (PE), urinary tract infection (UTI), deep vein thrombosis (DVT)	N/A
2	Elsamadicy et al. [19]	USA	92	Depressed cohort: 74.2 ± 6.4 years Non-depressed cohort: 71.7 ± 4.9,	Elective spinal surgery for correction of adult degenerative scoliosis	Koenig Depression Scale (KDS) ≥ 4	UTI, LOS, delirium, fever, infection, ileus, hypotension, hematoma, MI, PE, neurological injury (sensory deficit), DVT	6 months
3	Akins et al. [11]	USA	14,939	59 ± 13.4	Instrumented spinal fusions	N/A	Readmission	N/A
4	Chapin et al. [14]	USA	130	59	Lumbar spine surgery	Self-reported depression	LOS	3 and 12 months
5	Elsamadicy et al. [18]	USA	923	Depressed cohort: 61.9 ± 11.6 Non-depressed cohort: 61.0 ± 15.8	Elective spine surgery	Physician-diagnosed	Delirium, fever, hematoma, UTI, LOS, SSI, MI, PE, DVT, hypotension, ileus, infection, weakness, neurological injury (sensory deficit)	N/A
6	Held et al. [23]	Switzerland	401	Depressed: 72.2 ± 9.4 Non-depressed: 72.6 ± 8.2	Surgical treatment for symptomatic degenerative lumbar spinal stenosis (DLSS)	N/A	SSI, complications, reoperation	24 months
7	Holbert et al. [24]	USA	596	Depression or anxiety: 61.7 ± 13.3 No depression or anxiety: 61.2 ± 14.3	Decompression thoracolumbar surgery, fusion thoracolumbar surgery	N/A	LOS, readmission, complications, reoperation	12 months
8	Barreto Chang et al. [12]	USA	152	72 ± 5.4	Spine surgery for 3 h or longer	Prior clinical diagnosis	Delirium	N/A
9	Cui et al. [15]	China	242	79.8 ± 3.3	Posterior lumbar fusion surgery	Zung’s Self-Rating Depression Scale ≥ 53	Adverse event	90 days
10	Dietz et al. [16]	USA	173,519	56 ± 12	Laminectomy, laminotomy, discectomy, vertebrectomy, corpectomy, foreign body removal, and repair of vertebral fracture	N/A	Infection, SSI	24 months
11	Elsamadicy et al. [20]	USA	144,123	60.06 ± 14.59	Elective lumbar spine surgery (laminectomy discectomy fusion)	N/A	Readmission	90 days
12	Fineberg et al. [21]	USA	578,457	55.1	Lumbar decompression and lumbar fusion	N/A	Delirium	N/A
13	Gandhi et al. [22]	USA	647	55.7 ± 14.6	Posterior lumbar surgical procedures	N/A	Urinary retention	N/A
14	Huang et al. [25]	Taiwan	10,109	Depressed: 58.1 ± 13.7 Major Psychiatric Disorders: 59.5 ± 14.4 Control: 57.1 ± 15.2	Discectomy, laminectomy, and spinal fusion	N/A	Ventilator use, non-routine discharge (rehabilitation), readmission, LOS	N/A
15	Lee et al. [26]	USA	175	52.6 ± 16.4	Adult spinal deformity surgery	N/A	Readmission	24 months
16	Lee et al. [27]	USA	227	50.5 + 17.8	Spine deformity surgery	N/A	Readmission, reoperation	90 days
17	Mancuso et al. [28]	USA	532	56	Lumbar surgery	Geriatric Depression Scale (≥11 is a positive screen for depression)	Non-routine discharge (external services), LOS	12 weeks
18	Menendez et al. [29]	USA	5,382,343	54 ± 15	Major spine surgery (spinal fusion or laminectomy)	Preadmission diagnosis of depression	Adverse events, wound complications, mortality, LOS, non-routine discharge (rehabilitation)	N/A
19	Romero-Muñoz et al. [35]	Spain	282	57.3 ± 18.02	Elective spinal surgery	N/A	Neurological injury (SCI)	1–4 years
20	Schoell et al. [31]	USA	70,581	64.4	Lumbar spine surgery	Documented diagnosis of depression before surgery	Failed back surgery syndrome, neurological injury (nervous tissue damage and dural tear but not neurogenic bladder or cauda equina)	1 years
21	Susano et al. [33]	Portugal	716	73.6 ± 6.0	Spine surgery	N/A	Delirium	30 days
22	Wang et al. [34]	China	231	Total: 79.7 ± 3.5 Depressed: 79.3 ± 3.5 Non-depressed: 79.8 ± 3.4	Short-segment fusion surgery	Zung Depression Rating Scale (ZDRS)	LOS, reoperation, readmission, complications, delirium, SSI, MI, DVT, UTI, hematoma, pneumonia	2 years
23	Doi et al. [17]	Japan	121	Depressed: 65.8 ± 13.3 Non-depressed: 62.9 ± 14.4	Surgery for cervical compressive myelopathy	Hospital Anxiety and Depression Scale (HADS)	Complications	N/A
24	Boakye et al. [13]	USA	52,480	57 ± 12	Spinal fusion	International Classification of Disease, 9th/10th Revision (ICD-9/10)	LOS, complications, reoperation	1, 2, 5 years
25	Sivaganesan et al. [32]	USA	33,674	58 ± 14.5	Elective lumbar surgery	N/A	Readmission	90 days
26	Mummaneni et al. [30]	USA	608	63	Surgery for grade I spondylolisthesis	N/A	Non-routine discharge	30 days

**Table 2:** The pooled analyses evaluating the association between depression and postoperative medical and surgical complications. The bold estimates were statistically significant for each analysis.

Complication	Number of Studies	Pooled No. of Participants	Pooled Estimate (Odds in Depressed vs. Non-Depressed)	Heterogeneity Estimate (I^2^%)
Adverse events	6	5,435,818	1.14 (0.97–1.34)	59.3
Delirium	6	580,571	**1.92 (1.77–2.07)**	0
Deep vein thrombosis	4	1650	**3.72 (1.03–13.42)**	0
Fever	2	1015	**6.34 (1.63–24.71)**	33.4
Hematoma	3	1246	**4.70 (1.44–15.38)**	0
Hypotension	2	1015	**4.32 (1.88–9.95)**	48.8
Ileus	2	1015	2.99 (0.12–74.51)	87.5
Infection	3	1415	4.05 (0.60–27.30)	65.22
Myocardial infarction	4	1646	2.17 (0.64–7.35)	0
Pneumonia	2	631	2.38 (0.66–8.62)	0
Pulmonary embolism	3	1415	**3.79 (1.21–11.92)**	0
Neurological injury	2	1015	**6.02 (2.55–14.20)**	0
Surgical site infection	5	175,474	**1.36 (1.32–1.40)**	0
Urinary retention	3	1801	**4.63 (2.11–10.14)**	54.6
Urinary tract infection	4	1646	**1.72 (1.03–2.88)**	0
Weakness	2	1015	**6.52 (3.54–12.01)**	0

## Data Availability

The datasets analyzed to reach the conclusions of this review is available from the first authors (SA) and (AS) on reasonable request.

## Supplementary Materials

The following supporting information can be downloaded at: https://www.mdpi.com/article/10.3390/jcm13113247/s1, Table S1: Risk of bias and quality assessment using Newcastle-Ottawa scale. Forest plots for perioperative complications analyses are available in Figure S1: Adverse events, Figure S2: Delirium, Figure S3: Fever, Figure S4: Hematoma, Figure S5: Hypotension, Figure S6: Ileus, Figure S7: Urinary tract infection, Figure S8: Urinary retention, Figure S9: Infection/sepsis, Figure S10: Surgical site infection, Figure S11: Neurological injury, Figure S12: Pneumonia, Figure S13: Myocardial infarction, and Figure S14: Pulmonary embolism.
