# Supplementary material for: Impact of Depression on Postoperative Medical and Surgical Outcomes in Spine Surgeries: A Systematic Review and Meta-Analysis
Source: J Clin Med. 2024 May 31;13(11):3247. doi: 10.3390/jcm13113247 (PMC11172961; doi:10.3390/jcm13113247)
Supplement: Supplementary file 1 [file jcm-13-03247-s001.zip › jcm-2968699-supplementary.pdf]

Supplementary Table S1: Risk of bias and quality assessment using Newcastle-Ottawa scale.

Study	Q1	Q2	Q3	Q4	Q5	Q6	Q7	Q8	Total
Adogwa 2016	1	1	1	1	1	1	1	1	8
Elsamadicy 2017	1	1	1	1	2	1	1	1	9
Akins 2015	1	1	1	1	2	1	0	1	8
Elsamadicy 2017	1	1	1	1	2	1	0	0	7
Held 2022	1	1	1	1	2	1	1	1	9
Barreto Chang 2023	1	1	1	1	2	1	0	1	8
Chapin 2015	1	1	0	1	0	1	1	1	6
Cui 2023	1	1	1	1	2	0	1	1	8
Dietz 2023	1	1	1	1	1	1	1	1	8
Elsamadicy 2018	1	1	1	1	1	1	0	1	7
Fineberg 2013	1	1	0	1	2	1	1	1	8
Gandhi 2014	1	1	1	1	1	1	1	1	8
Huang 2021	1	1	1	1	2	1	1	1	9
Lee 2021	1	1	1	1	2	0	1	1	8
Lee 2022	1	1	1	1	2	1	1	0	8
Mancuso 2017	1	1	1	1	2	1	0	1	8
Menendez 2014	1	1	1	1	2	1	1	1	9
Romero-Muñoz 2020	1	1	1	1	1	1	1	1	8

Schoell 2019	1	1	1	1	0	1	1	1	7
Susano 2019	1	1	1	1	2	1	1	1	9
Wang 2023	1	1	1	1	2	1	1	1	9
Doi 2019	1	1	1	1	1	1	0	1	7
Boakye 2021	1	1	1	1	2	1	1	1	9
Sivaganesan 2018	1	1	1	1	2	1	1	1	9
Mummaneni 2020	1	1	1	1	2	0	1	1	8

Pooled results of the primary outcome for each complication (Figures S1–S14)

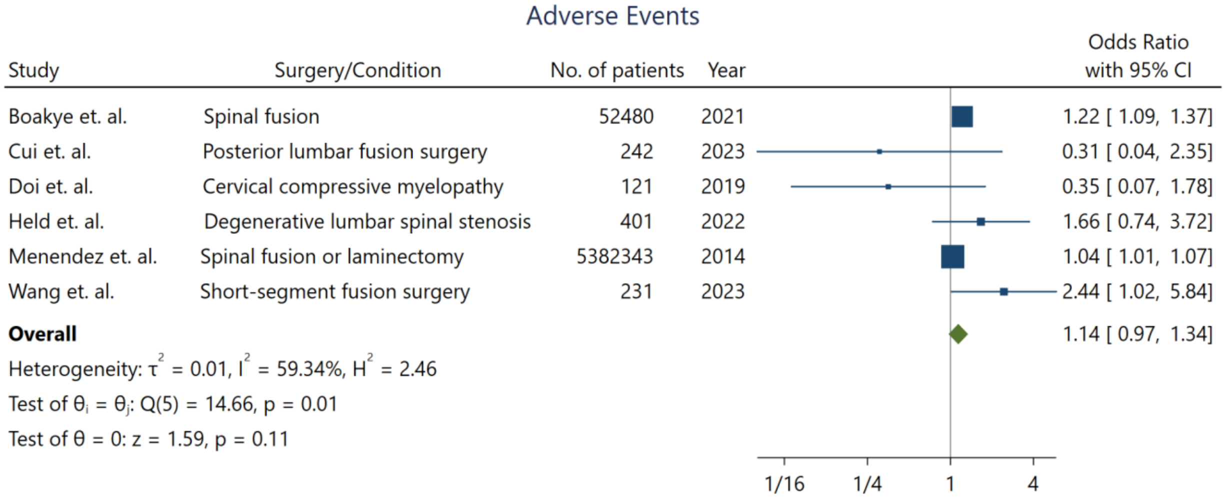

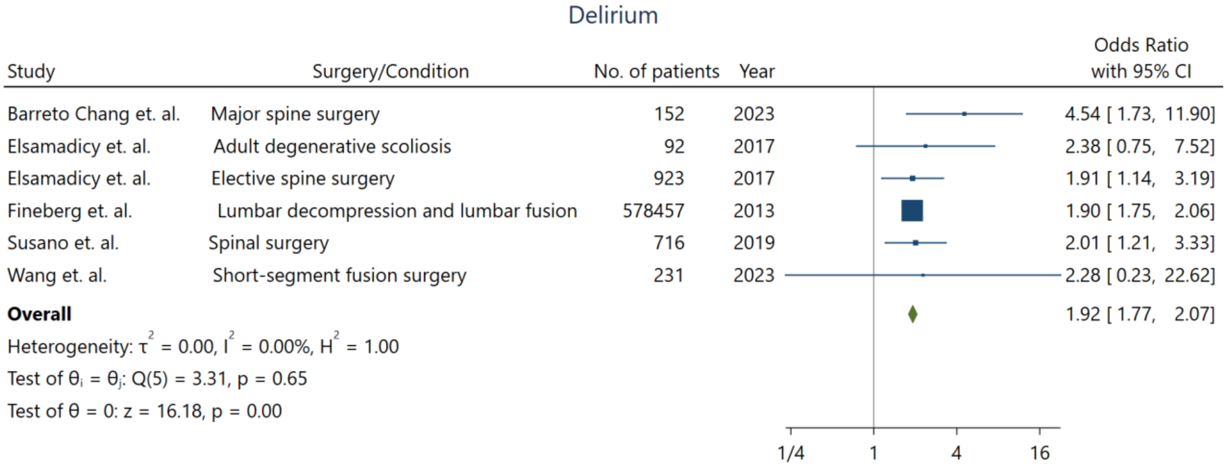

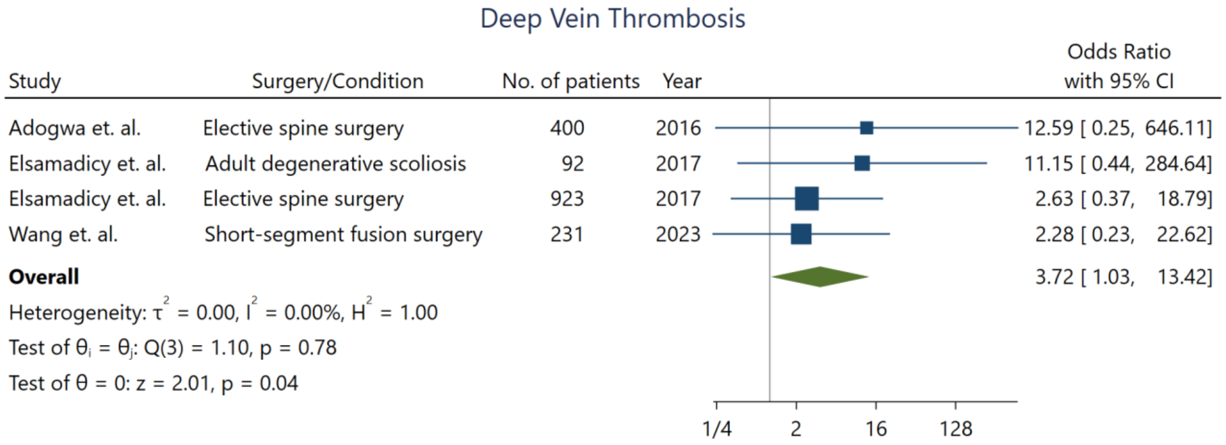

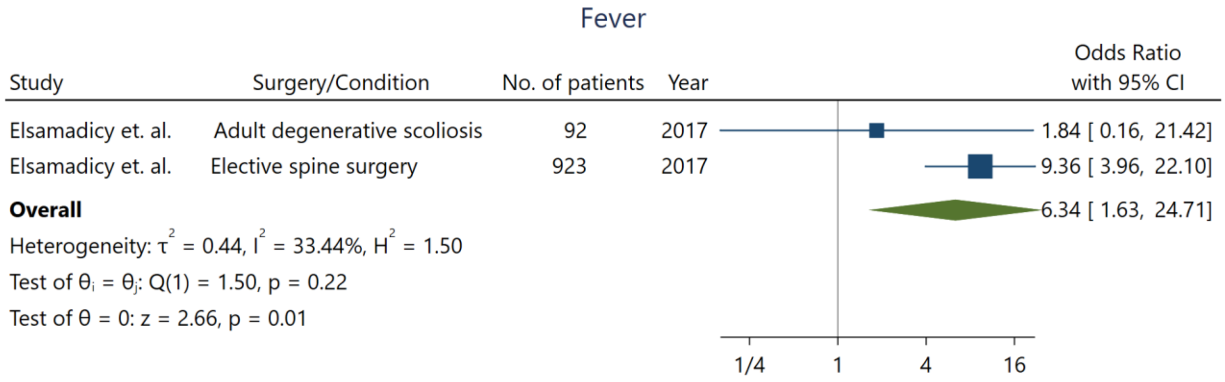
Fever
Study
Surgery/Condition
No. of patients
Year
Odds Ratio with 95% CI
Elsamadicy et. al. Adult degenerative scoliosis 92 2017 1.84 [0.16, 21.42]
Elsamadicy et. al. Elective spine surgery 923 2017 9.36 [3.96, 22.10]
Overall 6.34 [1.63, 24.71]
Heterogeneity: τ² = 0.44, I² = 33.44%, H² = 1.50
Test of θi = θj: Q(1) = 1.50, p = 0.22
Test of θ = 0: z = 2.66, p = 0.01
1/4
1
4
16

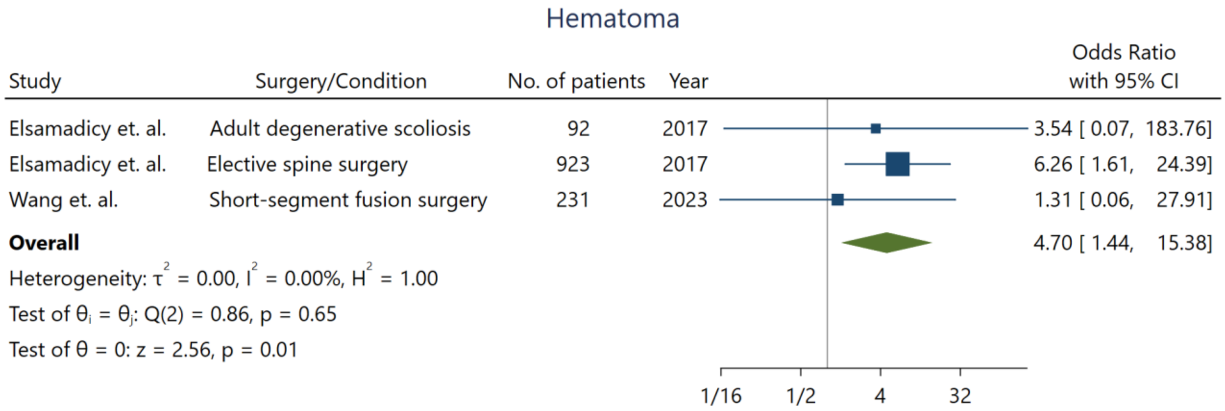
Hematoma
Study
Surgery/Condition
No. of patients
Year
Odds Ratio with 95% CI
Elsamadicy et. al. Adult degenerative scoliosis 92 2017 3.54 [0.07, 183.76]
Elsamadicy et. al. Elective spine surgery 923 2017 6.26 [1.61, 24.39]
Wang et. al. Short-segment fusion surgery 231 2023 1.31 [0.06, 27.91]
Overall 4.70 [1.44, 15.38]
Heterogeneity: τ² = 0.00, I² = 0.00%, H² = 1.00
Test of θi = θj: Q(2) = 0.86, p = 0.65
Test of θ = 0: z = 2.56, p = 0.01
1/16
1/2
4
32

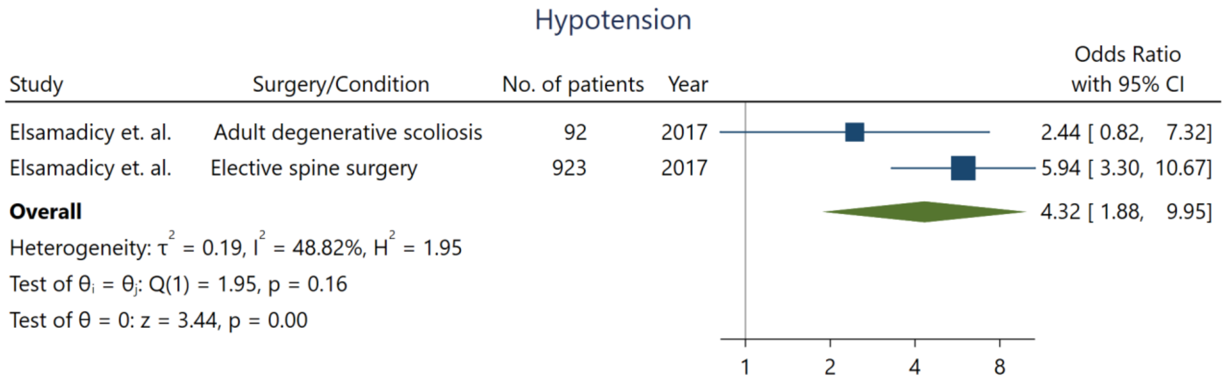
Hypotension
Study
Surgery/Condition
No. of patients
Year
Odds Ratio with 95% CI
Elsamadicy et. al. Adult degenerative scoliosis 92 2017 2.44 [0.82, 7.32]
Elsamadicy et. al. Elective spine surgery 923 2017 5.94 [3.30, 10.67]
Overall 4.32 [1.88, 9.95]
Heterogeneity: τ² = 0.19, I² = 48.82%, H² = 1.95
Test of θi = θj: Q(1) = 1.95, p = 0.16
Test of θ = 0: z = 3.44, p = 0.00
1
2
4
8

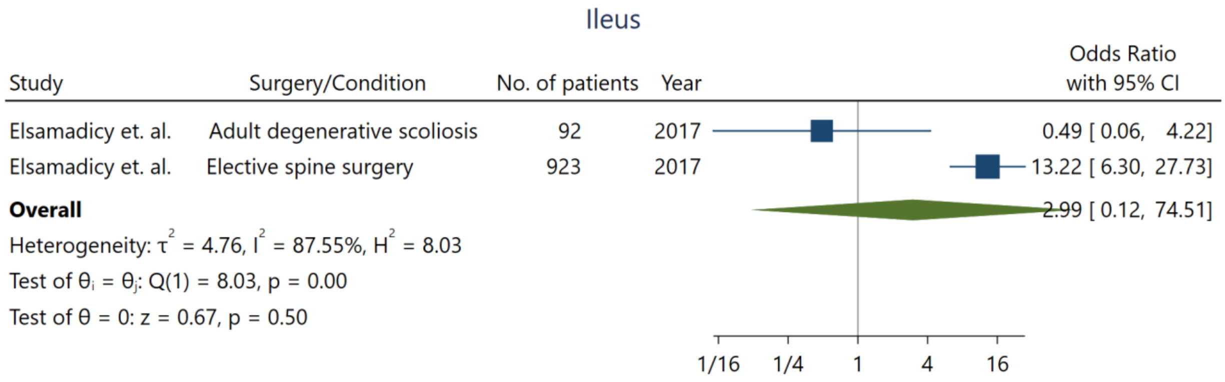
Ileus
Study
Surgery/Condition
No. of patients
Year
Odds Ratio with 95% CI
Elsamadicy et. al. Adult degenerative scoliosis 92 2017 0.49 [0.06, 4.22]
Elsamadicy et. al. Elective spine surgery 923 2017 13.22 [6.30, 27.73]
Overall 2.99 [0.12, 74.51]
Heterogeneity: τ² = 4.76, I² = 87.55%, H² = 8.03
Test of θi = θj: Q(1) = 8.03, p = 0.00
Test of θ = 0: z = 0.67, p = 0.50
1/16
1/4
1
4
16

Urinary Tract Infection

Study	Surgery/Condition	No. of patients	Year	Odds Ratio with 95% CI
Adogwa et. al.	Elective spine surgery	400	2016	1.82 [0.59, 5.57]
Elsamadicy et. al.	Adult degenerative scoliosis	92	2017	1.89 [0.32, 11.15]
Elsamadicy et. al.	Elective spine surgery	923	2017	1.59 [0.84, 3.00]
Wang et. al.	Short-segment fusion surgery	231	2023	3.43 [0.30, 39.05]
**Overall**				1.72 [1.03, 2.88]

Heterogeneity: $\tau^2 = 0.00$, $I^2 = 0.00\%$, $H^2 = 1.00$

Test of $\theta_i = \theta_j$: Q(3) = 0.39, p = 0.94

Test of $\theta = 0$: z = 2.06, p = 0.04

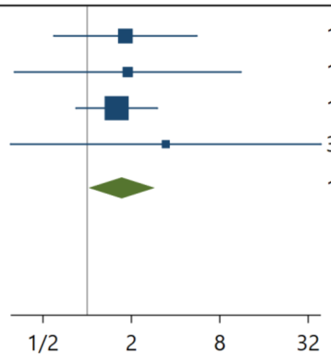

Urinary Retention

Study	Surgery/Condition	No. of patients	Year	Odds Ratio with 95% CI
Elsamadicy et. al.	Elective spine surgery	923	2017	7.18 [4.29, 12.03]
Gandhi et. al.	Posterior lumbar spine surgery	647	2014	2.51 [1.07, 5.89]
Wang et. al.	Short-segment fusion surgery	231	2023	4.71 [0.75, 29.46]
**Overall**				4.63 [2.11, 10.14]

Heterogeneity: $\tau^2 = 0.25$, $I^2 = 54.60\%$, $H^2 = 2.20$

Test of $\theta_i = \theta_j$: Q(2) = 4.30, p = 0.12

Test of $\theta = 0$: z = 3.83, p = 0.00

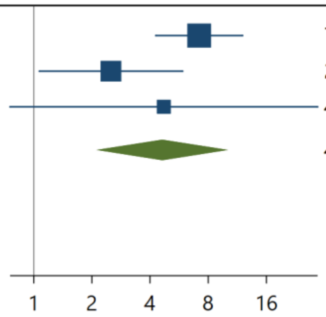

Infection/Sepsis

Study	Surgery/Condition	No. of patients	Year	Odds Ratio with 95% CI
Adogwa et. al.	Elective spine surgery	400	2016	1.89 [0.41, 8.74]
Elsamadicy et. al.	Adult degenerative scoliosis	92	2017	1.21 [0.12, 12.31]
Elsamadicy et. al.	Elective spine surgery	923	2017	30.07 [3.86, 234.13]
**Overall**				4.05 [0.60, 27.30]

Heterogeneity: $\tau^2 = 1.85$, $I^2 = 65.23\%$, $H^2 = 2.88$

Test of $\theta_i = \theta_j$: Q(2) = 5.63, p = 0.06

Test of $\theta = 0$: z = 1.43, p = 0.15

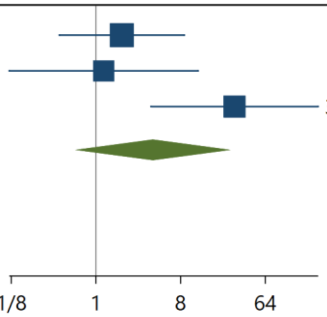

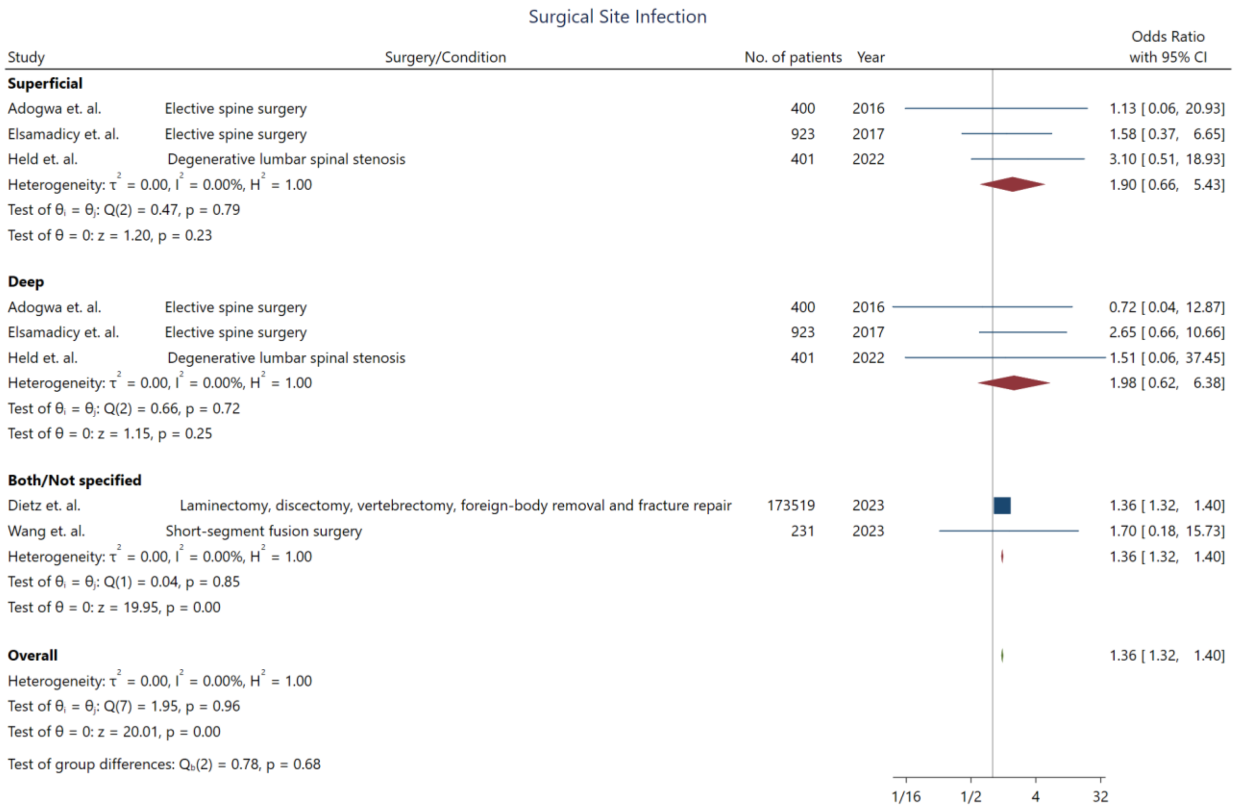

Surgical Site Infection
Study
Surgery/Condition
No. of patients
Year
Odds Ratio with 95% CI
Superficial
Adogwa et. al. Elective spine surgery 400 2016 1.13 [0.06, 20.93]
Elsamadicy et. al. Elective spine surgery 923 2017 1.58 [0.37, 6.65]
Held et. al. Degenerative lumbar spinal stenosis 401 2022 3.10 [0.51, 18.93]
Heterogeneity: τ^2 = 0.00, I^2 = 0.00%, H^2 = 1.00 1.90 [0.66, 5.43]
Test of $\theta_i = \theta_j$: Q(2) = 0.47, p = 0.79
Test of θ = 0: z = 1.20, p = 0.23
Deep
Adogwa et. al. Elective spine surgery 400 2016 0.72 [0.04, 12.87]
Elsamadicy et. al. Elective spine surgery 923 2017 2.65 [0.66, 10.66]
Held et. al. Degenerative lumbar spinal stenosis 401 2022 1.51 [0.06, 37.45]
Heterogeneity: τ^2 = 0.00, I^2 = 0.00%, H^2 = 1.00 1.98 [0.62, 6.38]
Test of $\theta_i = \theta_j$: Q(2) = 0.66, p = 0.72
Test of θ = 0: z = 1.15, p = 0.25
Both/Not specified
Dietz et. al. Laminectomy, discectomy, vertebrectomy, foreign-body removal and fracture repair 173519 2023 1.36 [1.32, 1.40]
Wang et. al. Short-segment fusion surgery 231 2023 1.70 [0.18, 15.73]
Heterogeneity: τ^2 = 0.00, I^2 = 0.00%, H^2 = 1.00 1.36 [1.32, 1.40]
Test of $\theta_i = \theta_j$: Q(1) = 0.04, p = 0.85
Test of θ = 0: z = 19.95, p = 0.00
Overall 1.36 [1.32, 1.40]
Heterogeneity: τ^2 = 0.00, I^2 = 0.00%, H^2 = 1.00
Test of $\theta_i = \theta_j$: Q(7) = 1.95, p = 0.96
Test of θ = 0: z = 20.01, p = 0.00
Test of group differences: $Q_b(2)$ = 0.78, p = 0.68
1/16
1/2
4
32

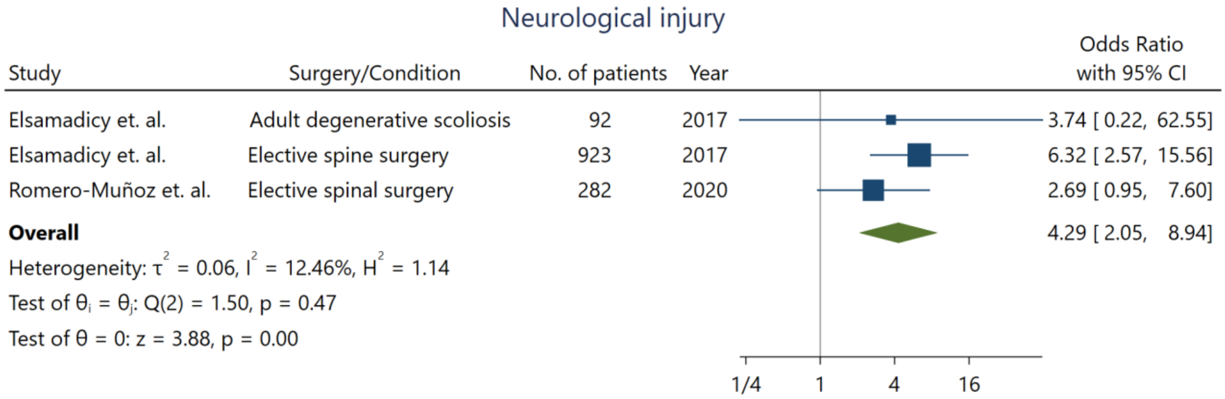

Neurological injury
Study
Surgery/Condition
No. of patients
Year
Odds Ratio with 95% CI
Elsamadicy et. al. Adult degenerative scoliosis 92 2017 3.74 [0.22, 62.55]
Elsamadicy et. al. Elective spine surgery 923 2017 6.32 [2.57, 15.56]
Romero-Muñoz et. al. Elective spinal surgery 282 2020 2.69 [0.95, 7.60]
Overall 4.29 [2.05, 8.94]
Heterogeneity: τ^2 = 0.06, I^2 = 12.46%, H^2 = 1.14
Test of $\theta_i = \theta_j$: Q(2) = 1.50, p = 0.47
Test of θ = 0: z = 3.88, p = 0.00
1/4
1
4
16

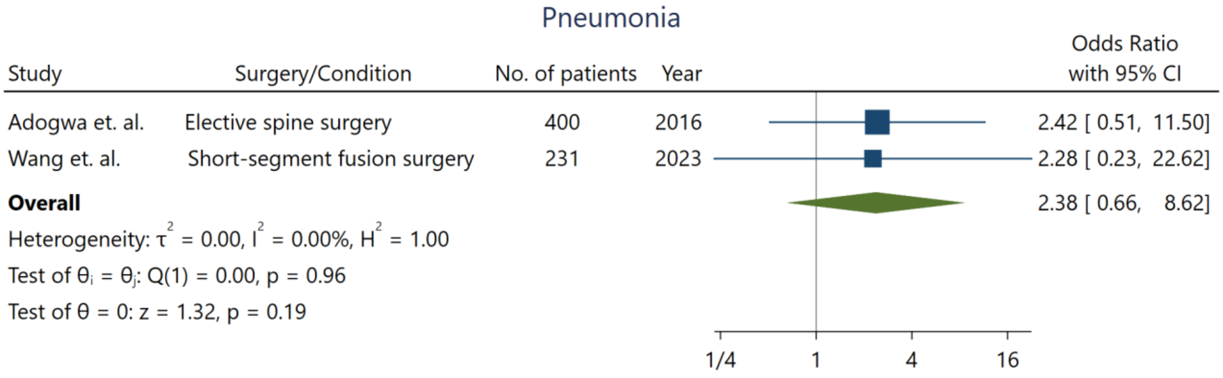
Pneumonia
Study
Surgery/Condition
No. of patients
Year
Odds Ratio with 95% CI
Adogwa et. al. Elective spine surgery 400 2016 2.42 [0.51, 11.50]
Wang et. al. Short-segment fusion surgery 231 2023 2.28 [0.23, 22.62]
Overall 2.38 [0.66, 8.62]
Heterogeneity: τ^2 = 0.00, I^2 = 0.00%, H^2 = 1.00
Test of $\theta_i = \theta_j$: Q(1) = 0.00, p = 0.96
Test of θ = 0: z = 1.32, p = 0.19
1/4
1
4
16

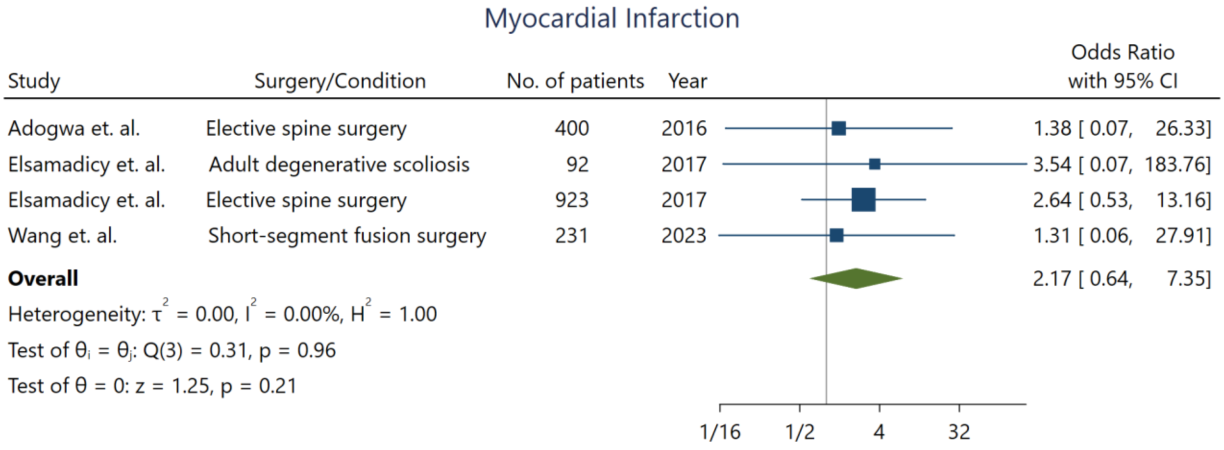
Myocardial Infarction
Study
Surgery/Condition
No. of patients
Year
Odds Ratio with 95% CI
Adogwa et. al. Elective spine surgery 400 2016 1.38 [0.07, 26.33]
Elsamadicy et. al. Adult degenerative scoliosis 92 2017 3.54 [0.07, 183.76]
Elsamadicy et. al. Elective spine surgery 923 2017 2.64 [0.53, 13.16]
Wang et. al. Short-segment fusion surgery 231 2023 1.31 [0.06, 27.91]
Overall 2.17 [0.64, 7.35]
Heterogeneity: τ^2 = 0.00, I^2 = 0.00%, H^2 = 1.00
Test of $\theta_i = \theta_j$: Q(3) = 0.31, p = 0.96
Test of θ = 0: z = 1.25, p = 0.21
1/16
1/2
4
32

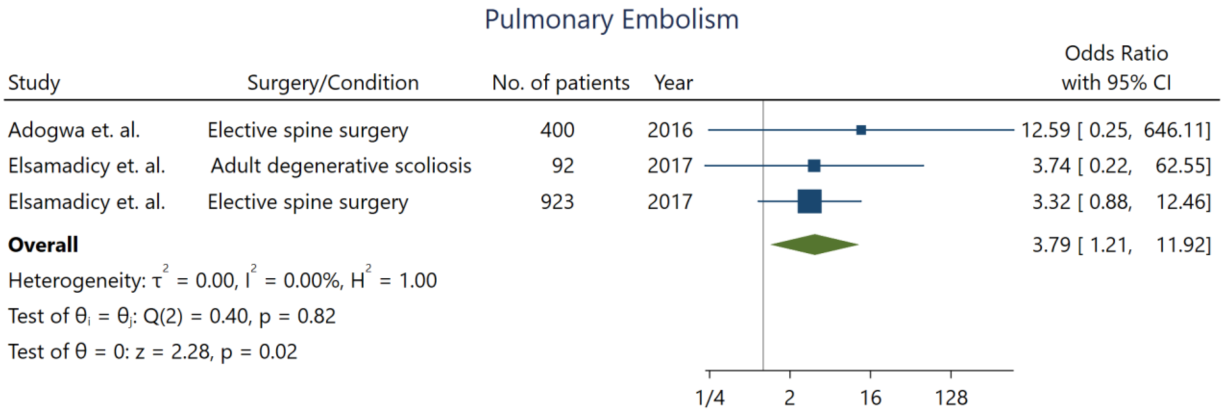
Pulmonary Embolism
Study
Surgery/Condition
No. of patients
Year
Odds Ratio with 95% CI
Adogwa et. al. Elective spine surgery 400 2016 12.59 [0.25, 646.11]
Elsamadicy et. al. Adult degenerative scoliosis 92 2017 3.74 [0.22, 62.55]
Elsamadicy et. al. Elective spine surgery 923 2017 3.32 [0.88, 12.46]
Overall 3.79 [1.21, 11.92]
Heterogeneity: τ^2 = 0.00, I^2 = 0.00%, H^2 = 1.00
Test of $\theta_i = \theta_j$: Q(2) = 0.40, p = 0.82
Test of θ = 0: z = 2.28, p = 0.02
1/4
2
16
128
